# Design of Silk-Elastin-Like Protein Nanoparticle Systems with Mucoadhesive Properties

**DOI:** 10.3390/jfb10040049

**Published:** 2019-11-12

**Authors:** Rachael N. Parker, Wenyao A. Wu, Tina B. McKay, Qiaobing Xu, David L. Kaplan

**Affiliations:** Department of Biomedical Engineering, Tufts University, Medford, OR 02155, USA; rachael.parker@tufts.edu (R.N.P.); annie.wu@tufts.edu (W.A.W.); tmckay333@gmail.com (T.B.M.); qiaobing.xu@tufts.edu (Q.X.)

**Keywords:** silk-elastin-like protein, mucoadhesion, transmucosal drug delivery, nanoparticles

## Abstract

Transmucosal drug delivery is a promising avenue to improve therapeutic efficacy through localized therapeutic administration. Drug delivery systems that increase retention in the mucosal layer are needed to improve efficiency of such transmucosal platforms. However, the applicability of such systems is often limited by the range of chemistries and properties that can be achieved. Here we present the design and implementation of silk-elastin-like proteins (SELPs) with mucoadhesive properties. SELP-based micellar-like nanoparticles provide a system to tailor chemical and physical properties through genetic engineering of the SELP sequence, which enables the fabrication of nanoparticles with specific chemical and physical features. Analysis of the adhesion of four different SELP-based nanoparticle systems in an artificial mucus system, as well as in in vitro cellular assays indicates that addition of mucoadhesive chemical features on the SELP systems increases retention of the particles in mucosal environments. The results indicated that SELP-based nanoparticles provide a useful approach to study and develop transmucosal protein drug delivery system with unique mucoadhesive properties. Future studies will serve to further expand the range of achievable properties, as well as the utilization of SELPs to fabricate mucoadhesive materials for in vivo testing.

## 1. Introduction

Mucus membranes that line the surfaces of many organs establish a protective barrier to microbial infection and unwanted foreign objects in the body through trapping and efficient removal. Consisting of up to 95% water, mucus layers are composed of hydrophilic glycoproteins known as mucins. Mucins comprise 3–5% of the mucus composition and are key to the barrier properties of mucus, providing structural integrity through physical entanglements and crosslinks [1]. Inorganic salts, lipids, carbohydrates, microbes, and enzymes are also present in the mucus. Although mucus barriers are essential to human health, their dynamic properties present a major challenge to drug delivery. Mucus turnover rates (i.e., time from initial mucus production to the time of complete replacement) range from minutes (e.g., in ocular mucosa [2]) to 1–2 days (e.g., in gastric and intestinal mucosa [3]), which leads to the rapid clearance of transmucosal and orally administered drugs.

Localized routes of administration include ocular, vaginal, nasal, buccal, and rectal mucosa, whereas oral administration relates to gastrointestinal mucosa. Mucoadhesive drug delivery systems that can adhere to the mucus layer and quickly penetrate the gel-like matrix offer a promising solution to the administer therapeutics. This strategy would provide a localized drug-reservoir allowing for prolonged release to the underlying submucosal tissue. This approach is also advantageous as it requires lower systemic doses in order to achieve an optimal therapeutic effect by favoring localized transmucosal delivery of the drugs [4,5]. Increased systemic bioavailability from prolonged drug residence times and improved epithelium absorption also represent promising advantages for transmucosal drug delivery. Localized and systemic delivery of therapeutics benefit from extended mucosal interactions, which leads to higher drug efficacy. However, major challenges to mucus retention and penetration stem from several physiological properties including the pH of the mucosal environment, activity of enzymes in the mucus, fluid volume, thickness of the mucus layer, and mucus turnover rates [6]. The efficiency of mucoadhesion depends on the interactions of the drug delivery system with the mucus.

Despite the diverse and dynamic microenvironment, the mucosal surface provides numerous physical and chemical features that can be exploited to increase mucoadhesion of carrier particles in drug delivery systems [6,7,8]. These approaches include particles with tailored hydrogen-bonding, electrostatic interactions, hydrophilicity, thiolation, and specific binding to mucins, as well as precise control over size and shape of the substrate [9,10,11,12,13,14,15]. Numerous mucoadhesive designs have been developed to improve retention of particles in mucus membranes, such as lipids [16], lectins [17], chitosan [18], synthetic polymers [19], and other biopolymers [9,20] in the form of adhesive tablets [21], micro- and nanoparticles [22], films [23], nanogels [24], and sprays [25]. For example, chitosan represents a widely utilized construction for developing mucoadhesive drug delivery systems. Mucoadhesive *N*-trimethyl chitosan chloride-coated poly (lactic-co-glycolic acid) nanoparticles improved retention in the intestinal mucus layer and increased bioavailability of insulin in diabetic rats [18]. However, these systems are often limited in tunability and generally require multi-step processes to impart desired chemistries or properties to the system. Additionally, approaches such as these often result in materials with limited utility because of the tedious nature of their synthesis. Our approach presents a novel route to develop mucoadhesion systems with a range of functionalities through modifications of the biopolymer sequence using genetic engineering, which increases the scope of materials that can be produced. Multifunctional platforms can enable highly specific and complex systems to greatly increase efficacy of transmucosal drug delivery. Further development of drug delivery systems that can be tailored to the mucosal environment and respond to local chemical differences is critical to the design of mucoadhesive particles with high drug efficacy.

Silk-elastin-like proteins (SELPs) have been extensively implemented in a variety of applications including biomaterials fabrication [26,27,28,29] and drug delivery systems [30,31,32,33]. Characterized by a repeating silk and elastin block copolymer structure, SELPs form a unique class of protein-based materials that are biocompatible, biodegradable, and highly tunable in terms of chemistry, size, and properties [26,31,32,34]. Through genetic engineering, modifications of the SELP amino acid composition and overall polymer size can be achieved, providing a route to tightly control the chemical and physical properties. Further utilization of these materials for the delivery of hydrophobic drugs to HeLa cells [31] and for self-assembled biomaterials demonstrate the range of properties achievable using SELPs [34]. The hydrophobic silk domain (GAGAGS) provides structural support and the hydrophilic elastin domain (GXGVP) enables modification of the chemical and stimuli-responsive properties through mutation of the variable sequence position “X”. We have previously reported the development of a library of SELP proteins by modification of the elastin sequence to generate SELPs with stimuli-responsive properties to pH, redox, temperature, electric field, and ionic strength, and utilized functional materials screening to isolate useful sequences from the library for further materials characterization [35]. The block copolymer structure of SELPs provides an additional design feature that enables future combinations of sequences with varying functionalities that will not affect the structure or inhibit material fabrication as reported in our previous work [36,37]. Thus, these proteins are useful candidates for designing mucoadhesive biopolymer systems because of their customizable physiochemical properties, where biocompatible SELP-based drug delivery systems that exploit favorable properties of mucosal membranes can be utilized.

The goal of the present study is to genetically engineer SELP-based materials and assess the effectiveness of SELP-based nanoparticles as mucoadhesive materials. We hypothesized that modifying the elastin domain to include specific charged amino acids and thiol-containing amino acid side chains (i.e., R,K,E,C for—arginine, lysine, glutamic acid, and cysteine) would provide improved mucoadhesion and drug retention in the mucosal epithelium. We designed four SELP systems that were then used to fabricate micellar-like nanoparticles incorporating the molecular probe, 8-anilinonaphthalene-1-sulfonic acid (ANS) as a model drug. This fluorescent compound allowed tracking of the nanoparticles in bio-mimetic mucus and in epithelial intestinal cell culture systems. The results support bioengineered SELP-drug-based delivery system designs as a useful strategy to explore toward future transmucosal drug delivery.

## 2. Results and Discussion

We have previously reported the design and production of recombinant SELP proteins with silk and elastin ratios of 1:4 (i.e., S2E8X), which contained arginine, lysine, glutamic acid, and cysteine in the variable position “X” of the elastin domain [35]. In the current study, we employed SELPs termed S2E8R, S2E8K, S2E8E, and S2E8C for the fabrication of micellar-like nanoparticles as model systems for investigating the utility of SELP proteins as mucoadhesive materials for drug delivery. The use of recombinant protein expression and purification methods allows for highly homogenous starting materials, where proteins are produced with yields from 20–40 mg/L following purification.

### 2.1. Characterization of ANS-Loaded SELP Nanoparticles

**Figure 1:**
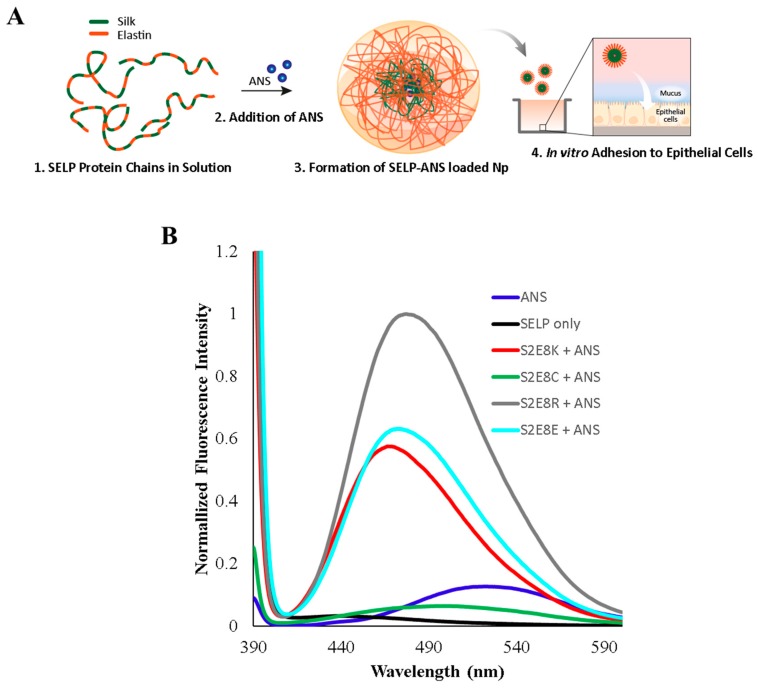
8-Anilinonaphthalene-1-sulfonic acid (ANS)-loaded silk-elastin-like proteins (SELP) assembly mechanisms and fluorescence characterization. (**A**) Assembly scheme shows ANS uptake in SELP micellar-like nanoparticles shown in four steps. 1. Depiction of free protein chains in solution. 2. Addition of ANS to induce micelle formation. 3. Depiction of the SELP-ANS loaded micelle-like structures. 4. Adhesion tests using in vitro cellular analysis. (**B**) Fluorescence characterization of SELPs before addition of ANS (black), after addition of ANS (S2E8K-red, S2E8C-green, S2E8R-grey, S2E8E-cyan), and free ANS in solution (blue). The black curve is representative of the protein samples without the addition of ANS. Spectra for each SELP sample is provided in Supplemental Information.

ANS is a hydrophobic, solvatochromic dye used to study conformational changes in protein structure [38], as well as to investigate drug loading in synthetic polymer systems [39]. Upon decreasing solvent polarity, a blue shift is exhibited in the ANS fluorescence spectrum, and the wavelength of maximum fluorescence intensity (λmax) changes from 545 nm to 470 nm. This shift results from a high affinity interaction of the dye with hydrophobic regions of the protein. Given the hydrophilic-hydrophobic block copolymer structure of the SELPs, ANS can be used as a hydrophobic trigger for micelle formation [31]. Specifically, the addition of ANS to SELPs in a polar solution facilitates assembly of micellar-like nanoparticles through interaction with the hydrophobic silk domain and creates a reporter system for analysis and tracking of the SELP nanoparticle system in subsequent studies presented here (Figure 1A).

Fluorescence spectroscopy measurements were used to assess the efficiency of SELP nanoparticle assembly upon addition of ANS. Following the previously described procedures using water as a solvent [31], ANS was added to each SELP solution and solutions were allowed to equilibrate prior to fluorescence measurements. Emission spectra corresponding to the aqueous SELP solutions before and after adding ANS, as well as an ANS only control, are displayed in Figure 1B. The emission spectrum of ANS only shows characteristic fluorescence (λmax = 545 nm) consistent with the hydrophobic dye in a polar environment. Additionally, each SELP protein excited at the same wavelength displayed minimal fluorescence (Figure 1B and Figure S1). Upon addition of ANS to the aqueous SELP solutions, all spectra exhibited a large shift in λmax, as well as a change in overall fluorescence intensity. Collectively, the SELP+ANS spectra are indicative of ANS in a nonpolar environment, which suggests encapsulation in the core of the SELP micelles. The λmax values (Table 1) show that S2E8K exhibits the largest shift in λmax with a λmax = 470 ± 2 nm compared to S2E8R = 479 ± 3 nm, S2E8E = 477 ± 2 nm, and S2E8C = 500 ± 4 nm. S2E8K and S2E8E display an increase in fluorescence signal approximately six-fold higher than that of S2E8C. However, S2E8R shows the greatest increase in fluorescence intensity corresponding to approximately ten-fold higher emission than that of S2E8C and approximately 1.6-fold higher than both S2E8K and S2E8E determined by comparing the fluorescence intensity value at λmax for each sample. These results suggest ANS encapsulation occurs with all protein sequences analyzed, and that S2E8R sequesters the highest concentration of ANS. This enrichment may be attributed to an increased affinity of the negatively charged dye and the arginine side chains found in the S2E8R construct indicating that favorable electrostatic interactions may increase uptake of ANS or enable stabilization of the dye in the micelle core. Interestingly, S2E8K and S2E8E show similar wavelength shifts, as well as similar intensity of emission despite the different charges associated with these proteins. However, the hydrophobic domains are the same, suggesting that the hydrophobic effect associated with the micelle formation is the strongest force contributing to the protein-dye association. This is further exemplified by the increase in fluorescence intensity shown by S2E8R. While S2E8R also contains the same hydrophobic domain, it appears to sequester more dye than S2E8K and S2E8E, indicating favorable electrostatic contributions to the dye sequestration. Although S2E8K also contains positively charged amino acid groups, the guanine side chain on the arginine groups of S2E8R likely forms a more stable complex with ANS than the amine group on the lysine from S2E8K given the more basic nature (i.e., pKa 12.5 compared to pKa 10.5) of the guanine group compared to the amine group resulting in a more stable cation and thus more stable ionic interaction. Additionally, S2E8C shows the lowest uptake of ANS. This finding may also be attributed to disulfide bond formation, which can reduce the ability of the SELP to sequester ANS. However, all SELPs evaluated demonstrate ANS uptake, which enables tracking of the system via fluorescence in future studies presented here.

To further probe encapsulation of ANS during SELP nanoparticle fabrication, DLS was implemented to measure the changes in hydrodynamic radii. Measurements of SELPs ranging in concentration from 0.1–1 mg/mL resulted in no observable differences in particle size as concentration was increased, indicating that aggregates do not form as concentration increases. The average hydrodynamic radii of the ANS-loaded SELP nanoparticles at 0.1 mg/mL (i.e., the concentration used for all presented studies) is 78 ± 1 nm for S2E8R, 102 ± 3 nm for S2E8K, 206 ± 3 nm for S2E8E, and 73 ± 4 nm for S2E8C (Table 1). Interestingly, S2E8E had an average particle size two-fold greater than all other SELP sequences. This size difference may be attributed to the negatively charged carboxyl groups of the glutamic acid side chains in the S2E8E sequence. Additionally, free protein chains in solution average around 4.5 nm [40] suggesting that for each sample, efficient micelle formation occurred. The polydispersity index (PDI) of less than 0.5 for all constructs, signifies relative homogeneity of nanoparticle sizes.

The proposed mechanism of assembly (Figure 1A) suggests that the hydrophobic effect is the driving force in formation of the SELP micelles and is consistent with previously investigated assembly of SELPs [34,40]. Collectively, fluorescence and DLS data indicate the formation of micellar-like nanoparticles and their efficient encapsulation of ANS for the four SELPs studied here, supporting further analysis of their mucoadhesive properties in the context of particle morphology delivery systems.

### 2.2. Evaluation of SELP Nanoparticle Mucoadhesive Properties

Because of the variability of mucus production by mucus-secreting cells in laboratory cultures, “bio-similar mucus” (BM) has been utilized as a controlled means to study the interaction of drug delivery systems with the mucus layer [7,41,42]. We implemented a 2D, cell-free BM model to investigate the interactions of the SELP nanoparticles in a mucus-rich environment. As previously described, SELPs containing arginine (R), lysine (K), glutamic acid (E), and cysteine (C) substitutes were selected for investigation as possible designs for mucoadhesive delivery systems, as these proteins contain charged groups and thiol groups resulting from the selected modification of the elastin “X” amino acid. Many studies have demonstrated the importance of electrostatic interactions in mucoadhesion [9,19,43,44]. Additionally, mucoadhesive nanoparticle drug delivery systems containing thiol groups have resulted in increased retention because of the propensity for disulfide bond formation with mucins containing thiol groups [44,45]. Therefore, we hypothesized that S2E8K, S2E8R, S2E8E, and S2E8C may have favorable chemistries to facilitate retention of the nanoparticles in the mucosal membrane.

**Figure 2:**
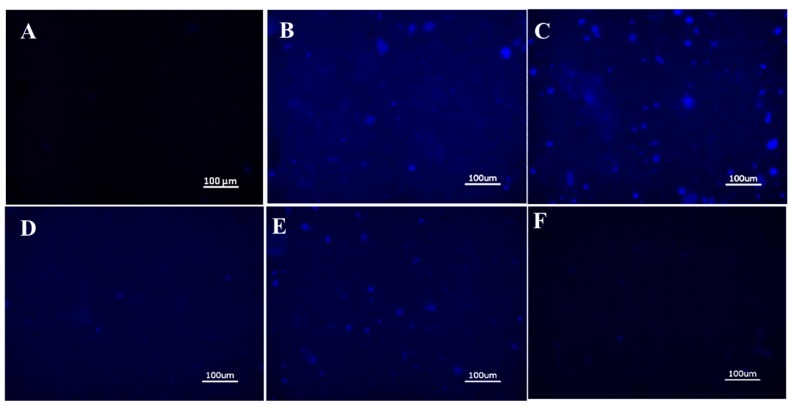
Fluorescence microscopy of bio-similar mucus SELP nanoparticle adhesion. Representative images of (**A**) untreated control, (**B**) S2E8R ANS-loaded nanoparticles, (**C**) S2E8K ANS-loaded nanoparticles, (**D**) S2E8E ANS-loaded nanoparticles, (**E**) S2E8C ANS-loaded nanoparticles, (**F**) Free ANS. All scale bars are 100 µm.

**Figure 3:**
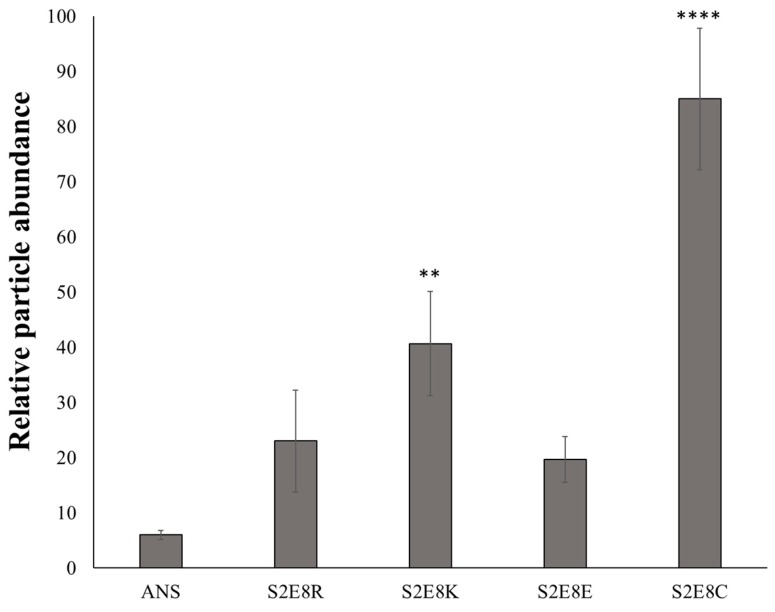
Quantification of SELP nanoparticle fluorescence microscopy images. Quantification of SELP nanoparticle retention in biosimilar mucus model. Counts generated by particle analysis using ImageJ software. Error bars represent standard deviation with n = 3. Statistical significance based on a one-way with Tukey’s multiple comparison shown. ** *p* < 0.01, **** *p* < 0.0001.

To test this hypothesis, BM was aliquoted into a standard 48-well cell culture plate followed by seeding of nanoparticles onto the mucus layer and subsequent incubation for 24 h, a time-frame consistent with gastrointestinal mucus turnover rates [3]. To reduce the retention of loosely bound particles, samples were washed with PBS and centrifuged to retain the mucus layer prior to imaging with fluorescence microscopy. Untreated samples and unbound ANS (i.e., free ANS in solution) were included, with the later providing an assessment of the diffusion rate of free dye molecules in the mucus layer (Figure 2A,F). Fluorescence microscopy images of the SELP nanoparticles indicated retention of nanoparticles in all samples, detected by imaging fluorescence emitted by encapsulated ANS molecules (Figure 2B–E). Free ANS was not detected in the nanoparticle untreated samples. DLS measurements indicate nanoparticles from 73 to 206 nm. However, it can be seen from the fluorescence images that large aggregates of several µm are formed throughout the mucus layer indicating accumulation of the particles. Particle analysis from ANS fluorescence (ImageJ) was used for quantitative assessment of nanoparticles retention in the mucus layer, demonstrating that S2E8C and S2E8K nanoparticles had the greatest retention (Figure 3). S2E8C nanoparticles showed the strongest affinity for the BM with a two-fold higher retention than S2E8K nanoparticles, and a three-fold and five-fold greater retention than S2E8R and S2E8E, respectively with S2E8E resulting in the lowest affinity of all samples in the BM model as determined by relative particle abundance (Figure 3).

As indicated by the lack of observed fluorescence in the free ANS samples and the localized areas of fluorescence observed in ANS-loaded nanoparticles, SELP nanoparticle encapsulation of the model fluorophore ANS was required for improving the retention of the compound in the mucus layer. Enhanced affinity of S2E8R and S2E8K to the BM over S2E8E likely resulted from favorable electrostatic interactions of the nanoparticles with the negatively charged mucin. These results were consistent with previous studies that have highlighted the importance of charge in maintaining drug system interactions with the mucosal membrane where positively charged nanoparticles lead to increased drug uptake [46,47,48,49]. The strongest affinity for the BM resulted from S2E8C, which contains cysteine as the variable amino acid. The presence of thiol groups in this nanoparticle system provided the ability to form disulfide bonds, likely leading to longer residence of the S2E8C ANS-loaded nanoparticles in the BM model.

### 2.3. In Vitro Cytotoxicity of ANS-Loaded Nanoparticles

**Figure 4:**
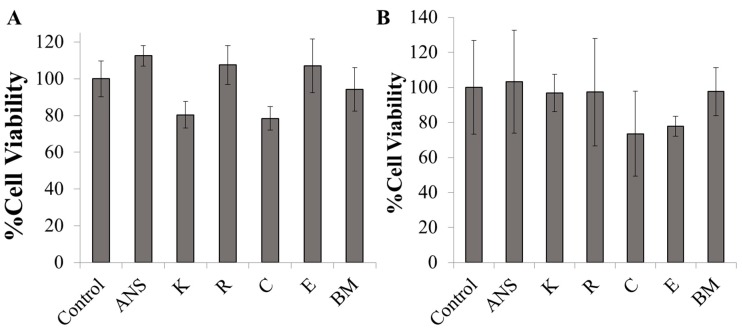
MTT assay results for (**A**) Caco-2 and (**B**) HT29-MTX following 24 h SELP nanoparticle incubation at 1 mg/mL. Error bars represent standard deviation with n = 3. No significant difference observed between control cells and treatment groups for Caco-2 or HT29-MTX based on a one-way ANOVA with Tukey’s multiple comparison.

To determine the biocompatibility of the SELP constructs, we evaluated the cell viability of Caco-2 and HT29-MTX cultures after incubation with the ANS-loaded SELP nanoparticles. No significant impact on cell viability was observed after 24 h when cells were dosed with nanoparticles up to 0.1 mg/mL, which was the highest concentration implemented in the in vitro assays (Figure 4A,B). SELPs have been previously shown to be biocompatible [31]. Therefore, our findings are consistent in showing that the SELP constructs developed in this study were non-toxic in vitro, thus supporting the applicability of a broad range of SELP-based systems for drug delivery.

### 2.4. Cellular Adhesion of ANS-Loaded SELP Nanoparticles

Further characterization of the adhesive properties of the SELPs was completed through examination of nanoparticle interactions with two different mucosal cell lines, Caco-2 and HT29-MTX. Retention of the nanoparticles in the mucus secreting epithelial HT29-MTX cells was compared to the Caco-2 cell line, which does not produce mucus. To evaluate the role of mucus production on adhesion of the ANS-loaded SELP nanoparticles in the cell layer, each SELP nanoparticle system was seeded on confluent Caco-2 and HT29-MTX cells and incubated for 24 h, after which the media were removed, and the cell layers washed with PBS and fixed with PFA. Untreated control cells were stained for the presence of F-actin using Alexa FluorTM 647 phalloidin and with nuclear DAPI stain to confirm integrity of the cell layer (Figure S2).

**Figure 5:**
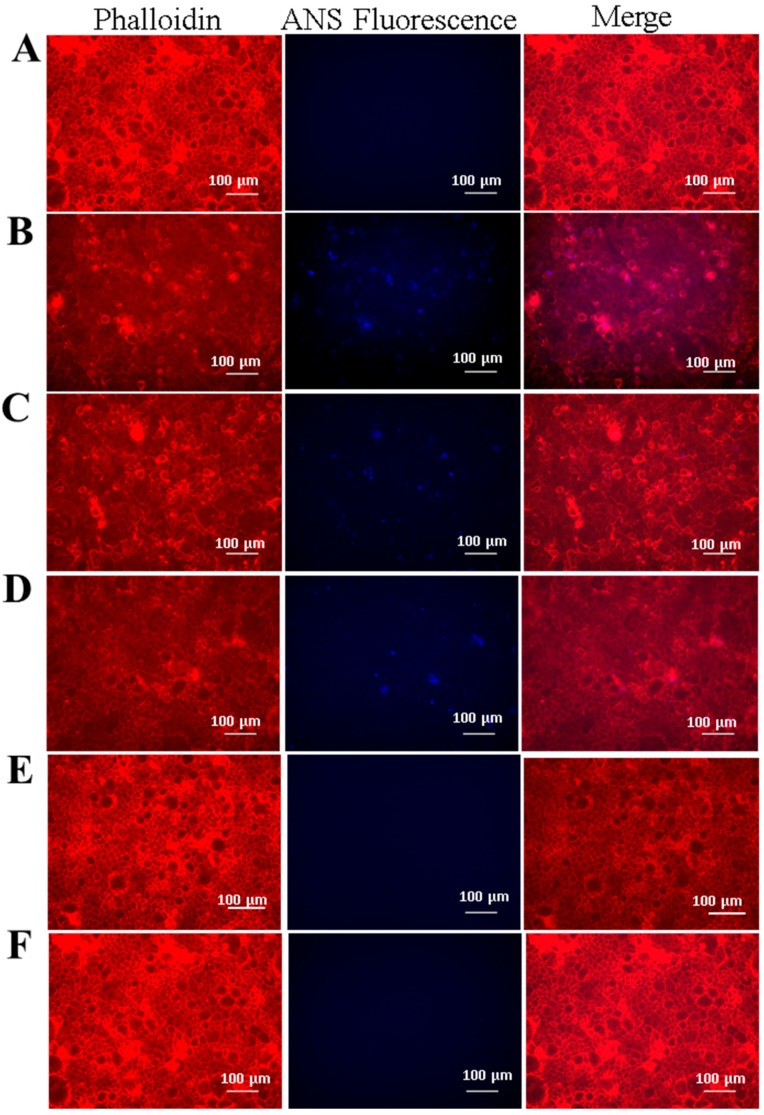
Fluorescence microscopy of Caco-2 cells seeded with ANS-loaded SELP nanoparticles. Representative images of (**A**) untreated control. (**B**) S2E8R ANS-loaded nanoparticles. (**C**) S2E8K ANS-loaded nanoparticles. (**D**) S2E8E ANS-loaded nanoparticles. (**E**) S2E8C ANS-loaded nanoparticles. (**F**) Free ANS. Cells are stained with Alexa FluorTM 647 phalloidin (F-actin, red) (λex = 560 nm and λem = 645 nm). ANS signal (blue) corresponds to λex = 360 nm and λem = 420 nm. All scale bars are 100 µm.

Fluorescence microscopy of the Caco-2 cells showed no retention of free ANS and no retention of S2E8C in comparison to the untreated cell layer (Figure 5A,E,F). Caco-2 cells do not produce mucus and it is therefore not unexpected that S2E8C would show diminished interactions compared to the BM analysis as the thiol containing cysteine groups are likely responsible for this result. S2E8R, S2E8K, and S2E8E displayed fluorescence signals observed from encapsulated ANS, suggesting retention of nanoparticles in the cell layer (Figure 5B–D). S2E8K and S2E8R nanoparticles were localized to the cell body and were characterized by discrete and uniform groupings suggesting possible endocytosis of the particles. However, this requires future validation to confirm. S2E8E appeared in larger amorphous clusters that indicated possible aggregation at the cell surface. SELP adhesion to hCSSCs was also assessed as an additional non-mucosal control cell line (Figure S3). Similar to the Caco-2 analysis, only S2E8K displayed retention in the cell layer, and no further adhesion was observed in the remaining systems.

**Figure 6:**
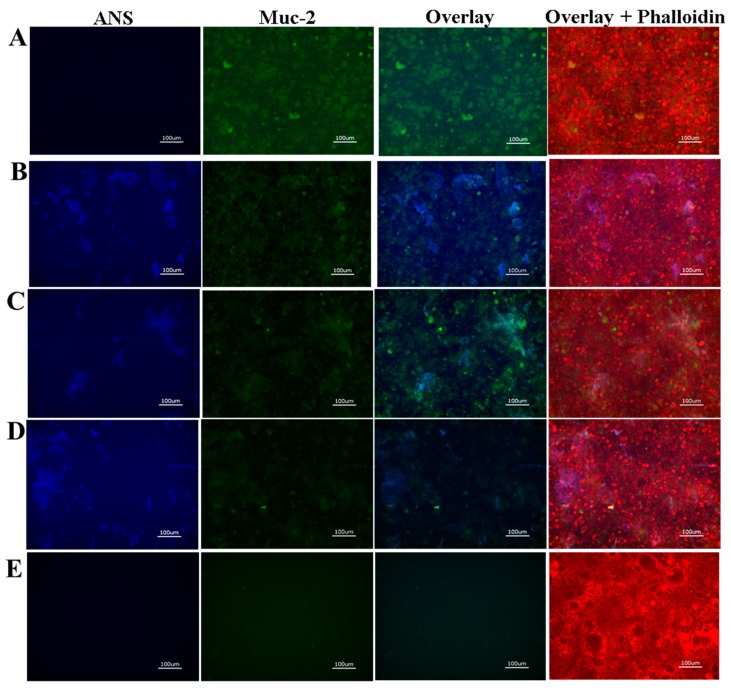
Fluorescence microscopy of HT29-MTX cells with ANS-loaded SELP nanoparticles. Representative images of (**A**) Free ANS. (**B**) S2E8R ANS-loaded nanoparticles. (**C**) S2E8K ANS-loaded nanoparticles. (**D**) S2E8E ANS-loaded nanoparticles. (**E**) S2E8C ANS-loaded nanoparticles. Cells are stained with Alexa FluorTM 647 phalloidin (F-actin, red) (λex = 560 nm and λem = 645 nm) and anti-Muc2 (green) (λex = 470 nm and λem = 525 nm). ANS signal (blue) corresponds to λex = 360 nm and λem = 420 nm. All scale bars are 100 µm.

**Figure 7:**
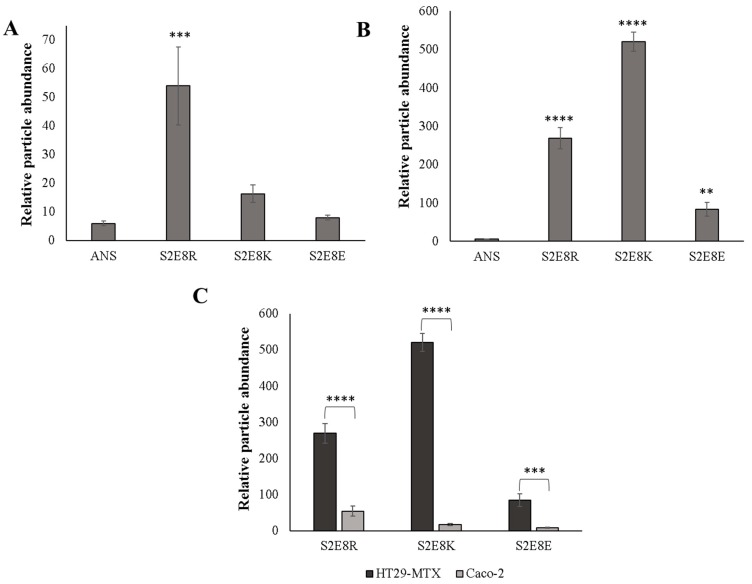
Quantification of SELP nanoparticle fluorescence microscopy images. (**A**) Quantification of SELP nanoparticle retention in Caco-2 cell model (**B**) in HT29-MTX cell model and (**C**) Comparison of SELP nanoparticle retention in HT29-MTX (dark grey) and Caco-2 (light grey) cells. Counts generated by particle analysis using ImageJ software. Error bars represent standard deviation with n = 3. Statistical significance based on a one-way or two-way ANOVA with Tukey’s multiple comparison shown in A–C or D, respectively. ** *p* < 0.01, *** *p* < 0.001, **** *p* < 0.0001.

To determine if the presence or absence of a mucus layer promoted increased drug retention in vitro, we assessed ANS-loaded SELP adhesion in a culture of HT29-MTX. In comparison, assessment of the HT29-MTX cells revealed adhesion of S2E8R, S2E8K, and S2E8E SELP samples to the cell layer, and as with the Caco-2 cells, no retention of the unbound dye (Figure 6). Additionally, S2E8C presented possible mucolytic properties based on the lack of mucus and nanoparticles signal observed in these samples. However, further studies are required to investigate this phenomenon. Determination of relative fluorescence abundance indicated a 10-fold increase in retention for S2E8E, 5-fold for S2E8R, and 32-fold for S2E8K, as determined by particle analysis from fluorescence imaging (ImageJ) (Figure 7A–C). To probe the role of mucus adhesion, we evaluated localization patterns of the SELPs with Mucin 2 (Muc2), an oligomeric glycoprotein responsible for mucus barrier formation [50]. HT29-MTX cells were stained with anti-Muc2 antibody to verify mucus production and accumulation, as well as to further assess the interactions of the nanoparticles with the cell layer. We observed high expression of Muc2 throughout the HT29-MTX culture consistent with the formation of a mucus layer. Regions of nanoparticle and Muc2 co-localization were evident suggesting that the SELPs may bind to the Muc2 glycoprotein, a favorable interaction that would likely improve drug retention in the presence of mucus (Figure 6B–D). Similar localization patterns between ANS-loaded nanoparticles and Muc2 were identified with little variability based on sequence suggesting that each variable amino acid may contribute to these binding interactions, but these mechanisms of adhesion require future studies to elucidate. These studies revealed an increased propensity of the nanoparticles for interaction with the HT29-MTX cell line consistent with the biomimetic mucus studies.

## 3. Materials and Methods

### 3.1. Biosynthesis of SELP Proteins

SELP constructs used in these studies are named according to the nomenclature S2E8X, corresponding to the sequence ((GAGAGS)_2_ (GVGVP)_4_ (G**X**GVP) (GVGVP)_3_) where **X** is either K, R, E, or C. SELPs of 57, 58, 63, and 65 kDa for S2E8K, S2E8C, S2E8R, respectively, were produced according to previously described procedures [31,34]. Briefly, expression vectors containing DNA sequences encoding for S2E8K, S2E8R, S2E8E, and S2E8C were transformed in BL21 (DE3) competent *E. coli* cells (New England Biolabs, Ipswich, MA, USA) and expressed under the T7 promoter. Following transformation, cells containing the expression plasmids were grown overnight for 16 h in 50 mL of Lysogeny Broth (LB) media at 37 °C with shaking at 250 rpm. Athena ES Hyper Broth (Fisher Scientific, Hampton, NH, USA) was then used to dilute the culture in a 1:100 ratio and cells were grown to an optical density (OD) of 0.8–1.0 before inducing protein expression with the addition of 1 mM isopropyl β-D-1-thiogalactopyranoside (IPTG). Proteins were expressed at 37 °C for 5 h followed by harvesting of cells by centrifugation at 5000 rpm for 15 min. Cells were then resuspended in PBS and stored at −20 °C until purification. All SELPs included an *N*-terminal histidine affinity tag and were purified using standard Ni-NTA affinity chromatography. Following purification, SELPs were dialyzed against deionized water for 3 days using Slide-A-Lyzer™ G2 Dialysis Cassettes, 10K MWCO (ThermoFisher, Waltham, MA, USA) before freeze-drying and storing for further use. Protein purity was assessed using sodium dodecyl sulfate polyacrylamide electrophoresis (SDS-PAGE).

### 3.2. Assembly of 8-Anilinonaphthalene-1-Sulfonic Acid (ANS)-Loaded Micellar-Like SELP Nanoparticles

ANS-loaded micellar-like SELP nanoparticles, herein referred to as SELP nanoparticles, were assembled as previously reported with minor modifications [31]. Briefly, concentrated ANS and SELP stock solutions made in water were mixed to achieve final concentrations of 80 µM and 1 mg/mL, respectively. Samples were incubated at 4 °C on a rotating mixer for 16 h followed by dialysis against deionized water using Slide-A-Lyzer™ G2 Dialysis Cassettes, 10K MWCO (ThermoFisher) for 1 day to remove unbound ANS molecules. For all experiments the stock solution was diluted to achieve a final SELP concentration of 0.1 mg/mL.

### 3.3. Dynamic Light Scattering (DLS)

The average particle size and size distribution of the SELP and ANS-loaded SELP micellar-like nanoparticles were measured using a DynaPro Titan dynamic light scattering (DLS) instrument (Wyatt Technology, Santa Barbara, CA, USA). Samples were filtered using a 0.45 µm syringe filter prior to analysis and all measurements were completed at 25 °C. SELP and ANS-loaded SELP nanoparticles were analyzed at 0.1–1 mg/mL. Herein we report DLS data as an average of three measurements per sample type.

### 3.4. Fluorescence Spectroscopy

Fluorescence of SELP and ANS-loaded SELP was monitored using a Hitachi F-4500 fluorescence spectrophotometer. Samples were prepared at concentrations of 0.1mg/mL and were filtered through a 0.45 µm syringe filter before analysis. Excitation of the samples occurred at 388 nm and spectra were recorded from 390 to 600 nm using 10 nm excitation and emission slits. The average fluorescence of three scans is reported for each sample.

ImageJ was used for determining the relative particle abundance for each sample based on the total fluorescence intensity in the DAPI channel (λex = 360 nm and λem = 420 nm). Images were converted into an 8-bit format followed by automatic threshold adjustment. The total counts per image were averaged over five representative regions of interest for three independent samples to determine the relative particle abundance per sample.

### 3.5. Cell Culture-Caco-2 and HT29-MTX Epithelial Cells and hCSSCs, and ANS-Loaded SELP Nanoparticle Adhesion to Epithelial Cell Monolayers

The HT29-MTX cell line was obtained from the Public Health England Culture Collections (Salisbury, Great Britain). The Caco-2 (CRL-2102) cell line was obtained commercially from ATCC (Rockville, MD, USA). Caco-2 and HT29-MTX cells were grown in Dulbecco’s Modified Eagle’s Medium (DMEM) (ThermoFisher) containing 10% v/v fetal bovine serum (FBS) (ThermoFisher) and 1% antibiotic-antimycotic (ThermoFisher). Cells were maintained at 37 °C with 5% CO_2_. Caco-2 and HT29-MTX cells were used from passage number 15–30 for all experiments. Human corneal stromal stem cells (hCSSCs) were provided by Jim Funderburgh (University of Pittsburg) following isolation from cadaveric corneal limbal tissue using established protocols [51]. hCSSCs (passages 4–5) were cultured in proliferation media composed of low glucose DMEM (Gibco, Grand Island, NY, USA), MCDB-201 (Sigma), 2% v/v FBS (Gibco), 10 ng/mL platelet-derived growth factor (PDGF), 10 ng/mL epidermal growth factor (EGF), 5 mg/mL insulin-transferrin-selenium (Gibco), 0.1 mM 2-phospho-L-ascorbic acid trisodium salt (Sigma-Aldrich, St. Louis, MO, USA), 10^−8^ M dexamethasone, and 50 μg/mL gentamicin (Life Technologies, Grand Island, NY, USA). After reaching 90% confluence, cells were differentiated to keratocytes [52] by culturing in advanced DMEM with 10 ng/mL fibroblast growth factor-2 (Sigma-Aldrich), 0.1 ng/mL transforming growth factor-β3, 50 μg/mL gentamicin, and 1% antibiotic-antimycotic (Gibco). Following 2 weeks of incubation, cells were seeded at a concentration of 1 × 10^5^ cells/cm^2^ and maintained in low serum media (Advanced DMEM (Gibco), 1% v/v FBS, 300 μg/mL ampicillin) until seeded with SELP nanoparticles at approximately 4 weeks. SELP and ANS-loaded SELP nanoparticles were prepared as described then diluted in culture medium to a final concentration of 0.1–1 mg/mL and seeded onto cells for a 24 h incubation period. The media was aspirated, and the cells washed with PBS prior to paraformaldehyde (PFA) fixation.

### 3.6. Immunohistochemistry

Cultures were fixed with 4% w/v PFA (Santa Cruz Biotechnology, Dallas, TX, USA) in PBS for 15 min at 25 °C for immunohistochemistry. After fixation, samples were washed three times with PBS and permeabilized with 0.1% v/v Triton-X-100 (Sigma, St. Louis, MO, USA) at 25 °C for 15 min followed by 3 PBS washes. Next, cells were incubated with a 3% w/v bovine serum albumin (BSA) blocking solution (ThermoFisher) for 1 h at 25 °C with agitation. After removing the BSA solution, cells were incubated with primary antibody solutions containing Alexa FluorTM 647 phalloidin (1:40, ThermoFisher, A22287) and anti-human Mucin2 (Mouse, 1:50, Abcam, ab11197) in PBS for 16 h at 4 °C with agitation. Primary antibodies were removed, and cultures were washed three times with PBS before addition of anti-Mouse IgG secondary antibody, Alexa FluorTM 488 conjugate (Donkey, 1:100, ThermoFisher, A21202), which was incubated with agitation for 1 h at 25 °C. All cultures were washed three times with PBS with agitation for 5 min at 25 °C prior to fluorescence microscopy. Control samples were also incubated with 4′,6-diamidino-2-phenylindole, dihydrochloride (DAPI) (ThermoFisher) at 1 µg/mL in PBS for 5 min at 25 °C and washed three times immediately before fluorescence microscopy.

### 3.7. Fluorescence Microscopy

All samples were imaged using the Keyence BZ-X700 fluorescent microscope (Keyence, Elmwood Park, NJ, USA). Images were collected using either a 10X or 20X objective. Filters used include DAPI, GFP, and Texas Red with excitation/emission wavelengths of 360 nm/420 nm, 470 nm/525 nm, and 560 nm/645 nm, respectively.

### 3.8. Preparation of Biosimilar Mucus and Adhesion of ANS-Loaded SELP Nanoparticles

Biosimilar mucus was prepared according to a published protocol [41]. All reagents were purchased from Sigma-Aldrich. Polysorbate 80 (0.163% w/v), phosphatidylcholine (0.18% w/v), and cholesterol (0.36% w/v) were dissolved in a solution of 137 mM NaCl, 10 mM 2-(*N*-morpholino)ethanesulfonic acid (MES), 1.3 mM CaCl_2_, and 1.0 mM MgSO_4_, pH 6.5. A second solution of BSA (3.1% w/v), mucin from porcine stomach (5% w/v), and polyacrylic acid (0.9% w/v) was prepared in the same buffer excluding NaCl. The solutions were mixed in a 1:9 ratio to obtain the necessary final volume. The pH was adjusted to 6.5, aliquoted in a 48-well plate in 500 µL aliquots and equilibrated at 4 °C for 16 h to form a thin layer on the bottom of the well plate. Following the equilibration step, the SELP nanoparticle samples were seeded on the biosimilar mucus at 0.1 mg/mL and incubated at 37 °C for 24 h. Samples were then washed three times with PBS before fluorescence imaging. For washing, PBS was added to each well followed by agitation at 25 °C for 5 min. The plate was then centrifuged at 5000 rpm for 5 min and the supernatant carefully removed. This was repeated for a total of three washes.

### 3.9. Cytotoxicity of SELP Nanoparticles and Biosimilar Mucus in Cell Culture Assays

Toxicity of the SELP nanoparticles and the biosimilar mucus was assessed using a Vybrant^®^ MTT Cell Proliferation Assay Kit (V-13154) (ThermoFisher) following the manufactures protocol. Briefly, Caco-2 and HT29-MTX cells were cultured in 24-well plates and incubated with either ANS-loaded nanoparticles (0.1 mg/mL) or biosimilar mucus for 24 h. Following incubation, the cell media was removed and replaced with phenol red-free media. A 12 mM stock of MTT 3-(4,5-dimethylthiazol-2-yl)-2,5-diphenyltetrazolium bromide (MTT) was prepared by dissolving 5 mg of MTT in 1 mL of sterile PBS. Ten microliter of the MTT stock solution was added to each well containing 100 µL of phenol-free media and incubated for 4 h at 37 °C. MTT was then solubilized by adding 50 µL of dimethyl sulfoxide (DMSO) with incubation at 37 °C for 10 min after which the absorbance of the samples was measured at 540 nm. An average of three absorbance measurements is reported.

### 3.10. Statistical Analysis

Statistical significance was determined based on a one-way or two-way analysis of variance (ANOVA), where appropriate, with alpha set to 0.05 (GraphPad Prism 7 for Windows, GraphPad Software, Version 7.03, La Jolla, CA, USA).

## 4. Conclusions

Transmucosal drug delivery systems are an important route for administration of a variety of drug classes. Development of mucoadhesive platforms that increase drug retention and absorption will greatly enhance the efficacy of transmucosal materials. The design flexibility afforded by genetic engineering technology allows for the formation of multifunctional, dynamic SELP nanoparticles. A major benefit of such systems is the ability to incorporate multiple functionalities into a single platform by implementing SELPs with unique physical and chemical properties into a composite material, unlike traditionally used materials that are often limited to a specific feature.

In the current report we detail the characteristics of model SELP-based mucoadhesive systems implementing four different protein sequences to fabricate micelle-like nanoparticles encapsulating ANS as a model compound and reporter. Through tailoring of the SELP primary sequence, we can generate chemically diverse SELP nanoparticle of precise size with high homogeneity. The utility of the SELP-based systems for mucoadhesion was explored by studying the affinity of the nanoparticles to an artificial mucus model, as well as by investigating the interactions of these systems in a mucosal cell model in vitro. The results indicated that the SELP-based approach provided a useful strategy to develop and study nanoparticles for transmucosal drug delivery in future studies.

Additional benefits of SELP-based designs include the ability to fabricate a variety of material formats, such as films, gels, and nanoparticles. Tailoring the sequence composition, structural properties, and stimuli-responsive features lend to the future development of SELPs to the design of novel drug delivery materials with controlled chemical and physical properties. However, additional studies are required to further probe drug release and efficacy using these systems. Future studies will investigate the use of engineered SELPs for fabrication of mucoadhesive materials.

## Figures and Tables

**Table 1:** SELP sequences and nanoparticle characterization. Molecular weight (MW), nanoparticle (Np) size, nanoparticle polydispersity (PDI), and maximum recorded wavelength (λmax) with highest fluorescence intensity are provided for each SELP sequence.

Name	Sequence	MW (kDa)	Np Size (nm)	Np PDI	Fluorescence λmax (nm)
S2E8R	GAGAGSGAGAGSGVGVPGVGVPGVGVP GVGVPGRGVPGVGVPGVGVPGVGVP	63	78 ± 1	0.2	479 ± 3
S2E8K	GAGAGSGAGAGSGVGVPGVGVPGVGVP GVGVPGKGVPGVGVPGVGVPGVGVP	57	102 ± 3	0.3	470 ± 2
S2E8E	GAGAGSGAGAGSGVGVPGVGVPGVGVP GVGVPGEGVPGVGVPGVGVPGVGVP	65	206 ± 3	0.2	477 ± 2
S2E8C	GAGAGSGAGAGSGVGVPGVGVPGVGVP GVGVPGCGVPGVGVPGVGVPGVGVP	58	73 ± 4	0.4	500 ± 4

## Supplementary Materials

The following are available online at https://www.mdpi.com/2079-4983/10/4/49/s1, Figure S1: Fluorescence spectra of SELP proteins. Figure S2: Representative fluorescence microscopy images of Caco-2 and HT29-MTX untreated cells. Figure S3: Representative fluorescence microscopy images of hCSSCs.
