# Supplementary material for: Design of Silk-Elastin-Like Protein Nanoparticle Systems with Mucoadhesive Properties
Source: J Funct Biomater. 2019 Nov 12;10(4):49. doi: 10.3390/jfb10040049 (PMC6963467; doi:10.3390/jfb10040049)
Supplement: Supplementary file 1 [file jfb-10-00049-s001.pdf]

Journal of
Functional Biomaterials

MDPI

Article

Design of Silk-Elastin-Like Protein Nanoparticle Systems with Mucoadhesive Properties

Rachael N. Parker, Wenyao A. Wu, Tina B. McKay, Qiaobing Xu and David L. Kaplan *

Department of Biomedical Engineering, Tufts University, 02155, United States; rachael.parker@tufts.edu (R.N.P.); annie.wu@tufts.edu (W.A.W.); tmckay333@gmail.com (T.B.M.); qiaobing.xu@tufts.edu (Q.X.)

* Correspondence: david.kaplan@tufts.edu; Tel.: +617-627-3251

Received: 5 October 2019; Accepted: 6 November 2019; Published: date

Supplementary Materials: Design of Silk-Elastin-Like Protein Nanoparticle Systems with Mucoadhesive Properties.

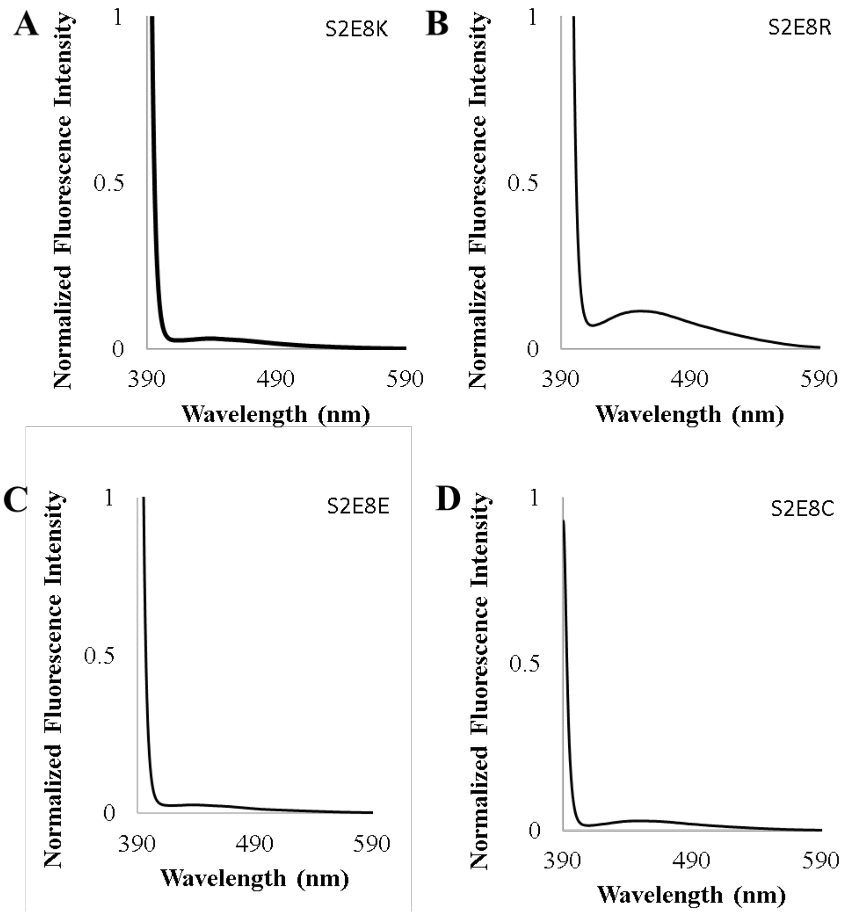

Figure S1. Fluorescence spectra of SELP proteins without ANS. (**A**) S2E8K (**B**) S2E8R (**C**) S2E8E (**D**) S2E8C. All spectra are reported as the average of 3 scans. Samples were excited at 388 nm and spectra were recorded from 390 to 600 nm.

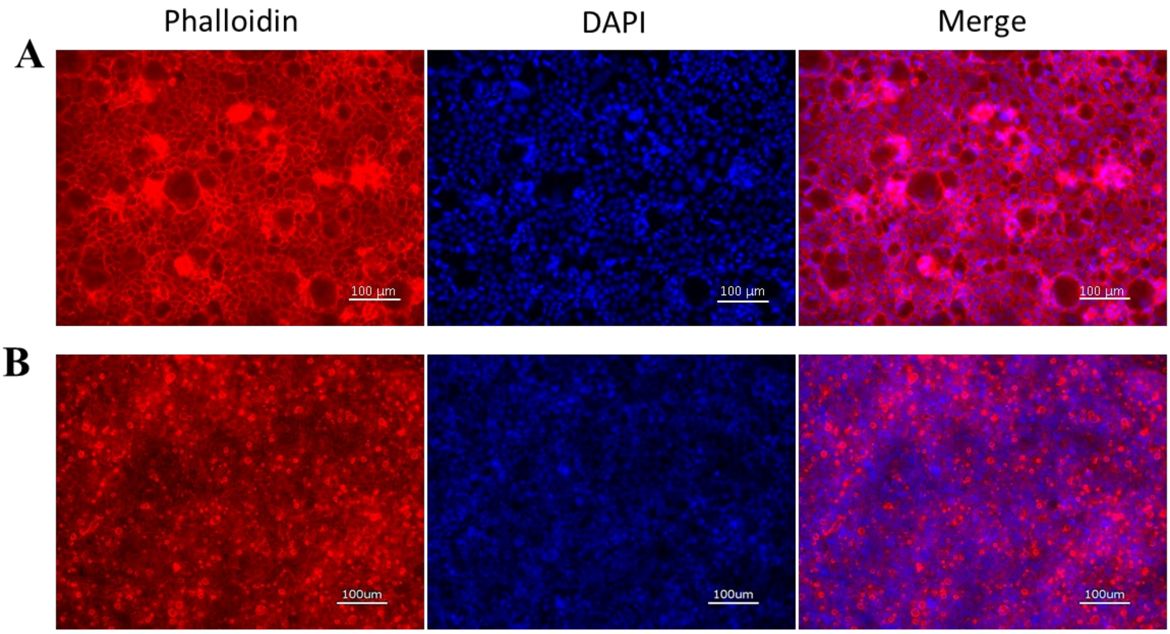

Figure S2. Representative fluorescence microscopy images of Caco-2 and HT29-MTX untreated cells. (**A**) Caco-2 (**B**) HT29-MTX. Samples were stained with Alexa Fluor™ 647 phalloidin (F-actin, red) and DAPI (blue) to determine the presence of a confluent cell layer. All scale bars are 100 µm.

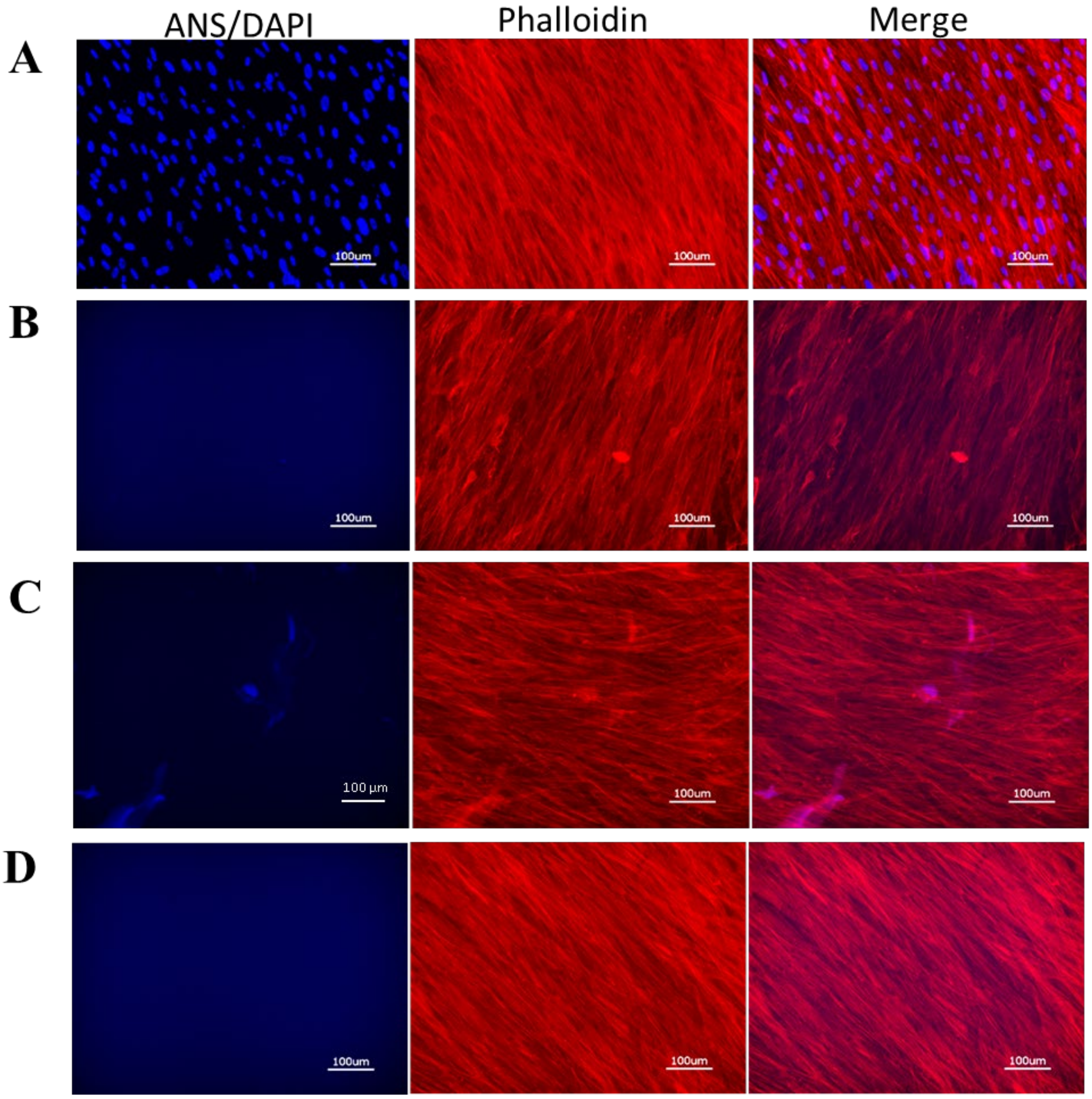

Figure S3. Representative fluorescence microscopy images of hCSSCs cells treated with SELP nanoparticle library. (**A**) Untreated control with nuclear DAPI stain (**B**) S2E8R (**C**) S2E8K and (**D**) S2E8E. Samples were stained with Alexa Fluor™ 647 phalloidin (F-actin, red) and DAPI (blue) to determine the presence of a confluent cell layer in panel A. Panels B–D show phalloidin (red) and ANS-loaded SELP nanoparticles (blue). All scale bars are 100 µm.
